# Medicine as a Career

**Published:** 1893-02

**Authors:** 


### Editorial, Etc.

MEDICINE AS A CAREER.—The current number of *The Forum* publishes a paper on "Medicine as a Career" from the pen of Dr. J. S. Billings. The article is interesting to the general reader and is particularly instructive to dentists as it raises some points pertinent to the dental profession. Following are some selections:

To the young man about to choose a professional career, medicine at this time offers opportunities for the employment of the highest mental faculties, for the increase of knowledge, for usefulness to the world, and for the attainment of true happiness, as no other profession presents. It is not meant by this to assert that it will certainly secure to its followers all or indeed any of these things, but that, given the same degree of intellect with a good preliminary education, the probabilities are that out of a thousand men taking up the study of medicine more will attain success than will do so among a similar number of young men of like character and attainments who devote themselves to theology, law, politics, or education.

In speaking of a medical career as a means of obtaining such success the word medicine is used in its broadest sense to include the study of the phenomena of human life—disease and death—the circumstances by which these can be influenced, and the practical application of the results to prevention as well as to cure.

A professional man is defined as one who professes or announces that he is in possession of special knowledge such as the great majority of men do not have, and that he is willing to furnish the benefit of his knowledge to his fellow men in the shape of advice or supervision of certain of their affairs. The three learned professions offer such advice and supervision with reference to men's souls, property, and bodies; but, as Mr. Evarts has remarked, the field of usefulness is more universal for medicine than it is for law or theology, since there are many who have no property to be cared for and there are some with regard to whose possession of souls there may be a question; but every body has a body, which at times is liable to require skilled management to avoid suffering and death and to enable it to do its work, although some bodies, it must be confessed, are hardly worth preserving.

Consider the practical usefulness of medicine. It is not merely physical pain in individuals that is lessened or averted. Its results extend far beyond the man whose disability is removed, whose pain is diminished, whose death is delayed, and who is thus enabled to go on with his share of the world's work; they affect the welfare and happiness of his family, of his associates, and, it may be, the interests of a nation, or of the world of science, of literature or of art.

The relative influence of heredity and of environment in the production of the defective, dependent, and dangerous classes of society, of the feeble-minded, the insane, the deaf-mutes, the vagrants, and the criminals, the means of preventing such production or of dealing with them after they have been produced, are all medical quite as much as they are sociological questions.

The professional educator, from the teacher in a common school to the head of a great university, has need of the information which the medical sciences are collecting with regard to the development of the organs of sensation, of memory, of comparison, and of judgment, which he is training for the coming generation, the men and women of the twentieth century; and with regard to the effects of variations in light, food, exercise, succession or order of studies, and many other things connected with school or college life upon the little masses of gray nerve substance which form the physical substratum of intellect, of emotions, and of morals. * * With a little change in certain cells of the cortical gray, a change which requires the use of the microscope to determine, the orator becomes speechless, the judge becomes a criminal, and the prudent man of business, the affectionate husband and father, the model citizen and pillar of the church becomes extravagant, unchaste, deceitful, and thus enters upon the first stage of a degeneration which, if unchecked, will make him a hopeless paralytic and a drivelling idiot.

I have said that a successful career brings to its pursuer the approval and friendship of those who best know his work, and this is pre-eminently true of practical medicine. * * Almost every reader of this paper knows some such man, in whose honesty of purpose, fidelity in keeping confidences, and readiness to undergo toil and trouble for the sake of his patients, all who know him have perfect confidence. * * Sweet are the confidence and friendship which come to him from those best qualified to judge of that part of his work which has been done rather for the benefit of the community, of science, and of the world than for individuals. These are for the most part, the members of his own profession whom he has helped. Some of them have been his immediate pupils; others, whom he may never see, have read his writings or have in other ways obtained help from his labors and express their appreciation of it in many ways.

The phrase "brother physicians" is one that applies especially in medicine, because for more than two thousand years in all civilized countries educated physicians have recognized each other as belonging to a brotherhood. * *

Thus medical men everywhere recognize the claim of a physician to advice and care in case of sickness or injury, and accept no fee for it. As Weir Mitchell says, "the physicians' guild is a world-wide guild, the only one."

It is necessary, in speaking of medicine as a career, to consider its demands as well as its inducements and these demands, for those who wish to secure such success as I have referred to, are for good mental capacity, for a long period of study, for much patience, for powers of physical endurance, for quick and keen sympathies, for honesty and for purity of thought, word and deed. In the hands of the physician are placed at times not only the issues of life and death, but of things more valuable than these. Not more than one man in a thousand can properly comply with these demands.

This done he will be ready to go to work, he will have some idea, though probably not an adequate one, of the things he does not know, and his advice and opinions will begin to be valuable. There is little cause to fear lest he do not find employment; there is always a place for a competent and trustworthy man, for one who can be depended on to work without supervision and to do more than the letter of his engagement calls for; and in laboratories, in hospitals, in medical schools, and in the broad field of practice there are such places waiting to-day for the men who have not yet been found for them.

This country is in no need of men possessing the diploma of Doctor of Medicine; it already has at least twenty thousand more of them than it requires or can properly support; but it does need several hundred, say a thousand, more of such properly-brained physicians as I have indicated, and I am quite sure that the people will be able to recognize them when they appear and will take proper care of their material interests.

My young friend whose attention I wish to direct to medicine as a career will have spent five years at a good intermediate school as a preliminary to entering the university.

which he does when he is about seventeen years old. He spends three or four years at the university, four years at a medical school, one and one-half in the hospital, and two years in travel and special studies. When, therefore, he is ready to begin work he will be about twenty-eight years old, and his education, living, books, etc., will have cost about eight thousand dollars from the time he entered the university. It can be done for less, but this is a fair average estimate. I am not considering medicine as a trade or looking at it from a commercial point of view.

